# Therapeutic Oligonucleotides Targeting Liver Disease: TTR Amyloidosis

**DOI:** 10.3390/molecules201017944

**Published:** 2015-09-30

**Authors:** Christoph Niemietz, Gursimran Chandhok, Hartmut Schmidt

**Affiliations:** Klinik für Transplantationsmedizin, Universitätsklinikum Münster, Albert-Schweitzer-Campus 1, Gebäude A14, D-48149 Münster, Germany; E-Mails: christoph.niemietz@ukmuenster.de (C.N.); gursimran.chandhok@gmail.com (G.C.)

**Keywords:** transthyretin, familial amyloid polyneuropathy, antisense oligonucleotide, small-interfering RNA, liver

## Abstract

The liver has become an increasingly interesting target for oligonucleotide therapy. Mutations of the gene encoding transthyretin (TTR), expressed in vast amounts by the liver, result in a complex degenerative disease, termed familial amyloid polyneuropathy (FAP). Misfolded variants of TTR are linked to the establishment of extracellular protein deposition in various tissues, including the heart and the peripheral nervous system. Recent progress in the chemistry and formulation of antisense (ASO) and small interfering RNA (siRNA) designed for a knockdown of TTR mRNA in the liver has allowed to address the issue of gene-specific molecular therapy in a clinical setting of FAP. The two therapeutic oligonucleotides bind to RNA in a sequence specific manner but exploit different mechanisms. Here we describe major developments that have led to the advent of therapeutic oligonucleotides for treatment of TTR-related disease.

## 1. Introduction

Therapeutic oligonucleotides bind via Watson-Crick hybridization to their molecular RNA targets that are known to be central for the pathomechanism of a disease, ultimately aiming at clinical improvement. Antisense oligonucleotides (ASO) and small interfering RNAs (siRNA) leading to RNA interference (RNAi) are amongst the two most widely used therapeutic oligonucleotides that were identified some decades ago [1,2]. One outstanding property of such oligonucleotides is the specificity of target binding which is much higher as compared to other drugs, commonly represented by small molecules or, more recently, by antibodies. Typically, the latter class of drugs can intercept with a receptor or an enzymatic reaction. In contrast, due to the property of oligonucleotides to target any given RNA, an outstanding number of possible targets can now be envisioned [3]. Supportive progress of the concept of therapeutic oligonucleotides stems from analyses derived from the Human Genome Project and large-scale transcriptome studies that have significantly broadened our understanding of how genetic information modifies disease. In this line, the previous dogmatic view on mRNA as the sole regulator of gene expression is now being replaced by a more complex picture. Various RNA species that are derived both from sense and antisense DNA strands giving rise to non-coding RNAs (ncRNAs), e.g., microRNAs, siRNA, and antisense RNAs, were identified to orchestrate gene expression as well as manifestation of disease [4,5]. The estimation of ~10,000 genes present in the human genome which are related to disease is therefore likely outnumbered by the various ncRNAs that are associated to disease. The continuing molecular characterization of disease therefore demonstrates the enormous potential of therapeutic oligonucleotides. Thus, therapeutic oligonucleotides significantly extend the range of clinical interventions, possibly supporting Ehrlich’s dream of a “magic bullet”, a drug so precisely targeted that its effectiveness is perfect and without any collateral damage. On a mechanistic view, different consequences can be achieved by therapeutic oligonucleotides. While downregulation of one Mendelian disease-causing gene is currently the most common application of therapeutic oligonucleotides, repair of disease-causing mRNA splicing events during the process of RNA maturation as well as upregulation of gene expression, e.g., by knockdown of RNA repressors, can be achieved [6,7,8]. Upregulation of a specific gene can be induced by oligonucleotides that inhibit the natural antisense transcripts of the gene, or interact with promoter/3′ UTR binding sites of repressor genes [8,9].

Since the first establishment of the proof-of principle in numerous preclinical models, direct transfer of therapeutic oligonucleotides to the clinic has, however, suffered from major technological hurdles. Different molecular barriers important for an efficient and safe targeting of the RNA have to be overcome. To achieve therapeutic efficacy, a high accumulation of the oligonucleotide in the target tissue as well as appropriate intracellular trafficking to the subcellular compartments harboring the enzymatic machinery for interaction with the target RNA have to be obtained [6,10]. Unwanted off-target effects that can be caused by immune responses directed to the oligonucleotide sequence itself or to other moieties residing in the drug formulation have to be critically monitored. It is therefore of no surprise that only a small fraction of oligonucleotides that passed first evaluations in preclinical models are further investigated in clinical studies. In the past couple of years, more than 100 ASO and RNAi-based therapies entered clinical trials [11,12,13]. From previous clinical studies, only fomivirsen (Vitravene; ISIS Inc., Carlsbad, CA, USA) and pegaptanib (Macugen; Pfizer/Eyetech Pharmaceuticals, Cedar Knolls, NJ, USA) were approved by United States Food and Drug Administration (FDA), the former in 1988 and the latter in 2004. Fomivirsen is an ASO, blocking translation of essential viral mRNA of cytomegalovirus (CMV), and was used for the treatment of immunocompromised patients, including patients with AIDS. Due to more effectice therapy for HIV patients, fomivirsen is no longer on the market. Pegaptanib, which binds to vascular endothelial growth factor (VEGF) rather than RNA represents an aptamer used for treatment of age-related macular degeneration. However, both drugs are delivered locally by intravitreal injection and therefore do not fully meet the breath of the many promises of therapeutic oligonucleotides, e.g., repeated systemic administration. The ups and downs in the development of therapeutic oligonucleotides for the market have resulted in some disillusionment and posing the question whether RNAi might be still alive [14]. In recent years however, continuous efforts from both academy and industry have obtained much progress for a refined development of therapeutic oligonucleotides allowing clinical use. In 2013, the first FDA approved therapeutic oligonucleotide, mipomersen, involving repeated and systemic administration, entered the market. Mipomersen (Kynamro; ISIS Inc.), is an ASO that targets apolipoprotein B (apoB) in patients with familial hypercholesterolemia [15]. This approval may now constitute an important nucleus for the field of therapeutic oligonucleotide.

The liver has evolved as a model target tissue where the concept of therapeutic oligonucleotides can be excellently evaluated [11]. Much of the attention to the liver as a model for therapeutic oligonucleotides is attributed to its central localization within the human circulatory system and a unique architecture of the organ. A significant number of genes involved in disease have been described for the liver that may allow therapeutic intervention by oligonucleotides [16]. Transthyretin (TTR), a human serum protein expressed by the liver, is the cause of familial amyloid polyneuropathy (FAP) [17]. Oligonucleotides directed against TTR mRNA are now under investigation in advanced clinical trials and attract major attention to the field, since the next clinical verification of the concept of therapeutic oligonucleotides seems to be close. This review will focus on recent advances in the ongoing clinical studies that involve ASO and siRNA directed against TTR.

## 2. TTR Amyloidosis

The phenotype, inheritance and organ specificity of TTR amyloidosis is highly different and three major clinical forms, TTR-FAP, TTR-FAC and TTR-SSA, have been described. TTR-FAP (OMIM: 176300) is a rare genetic disorder caused by mutations of the TTR gene in chromosome 18q12*.*1 and is inherited in an autosomal dominant manner [17]. First reports in 1952 by Corino de Andrade described a disease with characteristic paresis and impairment of neurological, digestive, and gastric function in several families from Portugal [18]. Subsequent histopathological studies revealed that the disease was associated with TTR deposits of amyloid in various organs and tissues [19]. The first description of mutated TTR protein was in 1983 and identified an amino acid substitution of methionine for valine at position 30 of the mature polypeptide [20]. This mutation, found in Portuguese patients, was subsequently shown to be the most prevalent form of TTR-FAP worldwide. While the frequency of Val30Met mutation is especially high in endemic areas of Portugal, Sweden and Japan, other local predominant mutations were identified around the world [21,22,23,24]. To date, more than 130 disease-causing TTR mutations have been described.

The homotetrameric TTR protein (about 55 kDa) comprises four subunits of the 127 amino acid protein and functions primarily as a carrier of retinol (vitamin A) and of thyroxine (T4) [17]. TTR is one of the most predominant serum proteins (160–350 mg/L) and has a half-life in blood of about 48 h [25]. Although having diverse biological functions, TTR knockout in mice was followed by viable offsprings without overt disease that, however, show reduction of circulating T4 and vitamin A levels [26,27] suggesting that in the absence of TTR major biological functions of the carrier can be compensated. TTR is predominantly produced in the liver (>95%) and secreted into blood with only a small amount of protein synthesized in the choroid plexus and retinal pigment epithelium.

**Figure 1:**
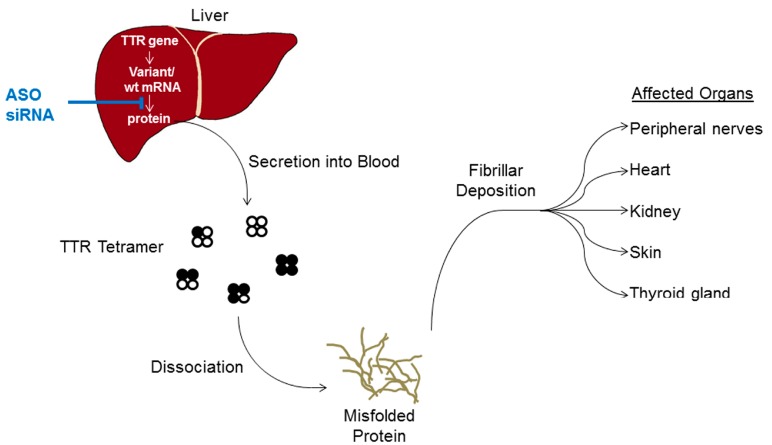
Schematic illustration of FAP amyloidosis. Wild-type and mutant TTR (open and closed circle, respectively) are secreted by the liver into blood as a tetrameric protein that is unstable when comprising mutant protein. Tetramers dissociate into monomers which misfold and aggregate. Deposition of TTR fibrils is observed in peripheral tissues and organs. In order to treat FAP, antisense oligonucleotides (ASO) and small-interfering RNAs (siRNA) that target TTR mRNA in the liver are currently evaluated in clinical studies.

The pathogenic process that is involved in TTR amyloid deposition is believed to be attributable to a decreased thermodynamic stability of tetramers containing mutant subunits that are prone to dissociation into dimers and monomers (Figure 1) [28]. Of note, tetramers found in the serum can be composed of a variable number of both wild type and mutant TTR. *In vitro*, TTR monomers can undergo partial unfolding allowing self-aggregation and ultimately polymerization into fibrils causing amyloidosis [29]. The exact molecular mechanism of TTR amyloid deposition in the tissues and its relevance to induce pathogenesis is however not completely understood. *In vitro* studies of tetrameric TTR dissociation demonstrated that even wild-type TTR tetramers can undergo conformational changes into amyloidogenic forms [30]. While extracellular deposits contain 30%–40% wild type monomers and also TTR cleavage products [24,31], the role of wild type TTR monomers to initiate or modulate plaque formation *in vivo* has to be further studied. It is of interest that in a different form of TTR-related disease, senile systemic amyloidosis (SSA), no mutation of TTR has been observed. SSA is highly common in males and predominantly affects the heart at an age of >60 years [32,33]. Plaques were found in 25% of a Finnish cohort above 85 years indicating that SSA is of high importance for countries with a high proportion of the elderly population [34]. At least in SSA, in the absence of mutant TTR, wild type TTR protein may therefore add to pathogenesis.

The phenotype of TTR-related amyloidosis is diverse and the extracellular deposition in various tissues, mainly peripheral neurons, gastrointestinal tract and the heart, shows plaques comprising TTR. TTR-FAP is associated with severe disease, including sensory, motor and autonomic neuropathy, and also cardiomyopathy is observed with a large continuum of disease symptoms in patients. Life expectancy of FAP patients is significantly reduced. Death is mostly observed after 5–15 years from onset of first symptoms. Cardiomyopathy-related amyloidosis is most frequently observed in patients of Danish and African ancestry having Leu111Met and Val122Ile mutations, respectively [35,36]. This clinical manifestation which usually has no pronounced neuropathy is known as familial amyloid cardiomyopathy (FAC). Diagnosis of TTR-related disease can therefore be challenging due to the variety of symptoms. Identification of amyloid deposits is confirmed by tissue biopsy usually from the skin. Light microscopy of tissues derived from the affected organ show an apple-green birefringence after congo red staining of fibrils. Isoelectric focusing (IEF) can be used to identify the TTR variant in serum. In case of the two hereditary forms of TTR amyloidosis, FAP and FAC, DNA sequencing is used to confirm the disease. While in some patient cohorts individual TTR mutations are linked to a major set of clinical manifestations, a direct genotype to phenotype correlation is not observed [37]. Patients in cohorts from Portugal with the Val30Met mutation start to develop symptoms at the age of 30 years and are classified by signs of autonomic and peripheral neuropathy [38,39]. However, Swedish patients, having the same genotype, show a later age at onset of about 56 years indicating that other factors influence disease [21]. Apart from polyneuropathy, some patients with Val30Met mutation can develop cardiomyopathy of late onset [40].

In a procedure called domino liver transplantation, where livers from FAP patients are transplanted, TTR related disease mechanism were observed after some years corroborating the disease-causing property of mutant TTR [41]. Orthotopic liver transplantation (OLT) to FAP recipients is used as a clinical option to treat the disease. OLT inhibits novel synthesis of mutant TTR protein by the liver and reduces the total mutant protein to about 1% of pre OLT serum values [42]. Liver transplantation of FAP patients having the TTR Val30Met mutation shows the best prognosis, particularly when recipients are at an early stage of the disease. However, a stabilization of disease rather than a complete remission can be achieved in most patients with a slow but ongoing progression of neuropathy [43]. Whether mutant TTR of choroid plexus (<5% of total TTR synthesis) that can pass the blood brain barrier is causative, remains to be investigated [44]. In the case of TTR amyloidosis with prominent heart involvement, it has been shown that wild type TTR itself accumulates on existing amyloid plaques after transplantation [45]. In patients diagnosed with FAC or FAP with prominent heart involvement, liver transplantation is therefore contraindicated since progression of cardiac disease is ongoing and would probably be exacerbated by wild type TTR synthesized by the donor liver [46,47]. As a consequence, OLT is not an option for almost two-thirds of FAP patients either due to age or advanced disease.

Considering that tetramer dissociation is a critical aspect of fibril formation, previous research has put some emphasis on the development of small molecules counteracting this process via tetramer stabilization. The hydrophobic thyroxine binding sites of the TTR were targeted in these approaches [48,49]. Diflunisal (Merck and Co., Inc., Kenilworth, NJ, USA), an anti-inflammatory drug originally developed in 1971, has been recently evaluated in a double-blind, placebo controlled clinical study for FAP treatment [50]. Tafamidis (Pfizer, Inc., New York, NY, USA) is currently the only approved drug for treatment of TTR amyloidosis. Tafamidis was introduced in 2011 in various European countries for treatment of TTR amyloidosis in adult patients with stage 1 polyneuropathy [51,52,53]. However, as opposed to therapeutic oligonucleotides, Tafamidis interferes after protein translation and does not reduce overall serum TTR. Specific inhibition of TTR mRNA by ribozymes as observed by *in vitro* studies seems to represent an alternative therapeutic option [54,55].

## 3. Molecular Mechanism of Antisense Oligonucleotides

Since the first description of gene inhibition resulting from a 13-mer ASO against Rous sarcoma viral RNA [56], further research has explored the remarkable potential of synthetic oligonucleotides. ASO are short nucleic acids, mostly composed of ssDNA of ~12–20 nucleotides, that bind to target RNA via Watson-Crick base pairing [57]. Depending on the location of ASO binding to regions of the RNA target, several mechanisms of gene editing can be obtained. In the cytoplasm, ASO can bind to the starting AUG codon of the mRNA and consequently inhibit the formation of the translation initiation complex by formation of steric barriers [58]. In the nucleus, ASO binding to pre-mRNA close to the splicing sites can affect RNA processing [59].

### 3.1. RNase H Mediated Cleavage

**Figure 2:**
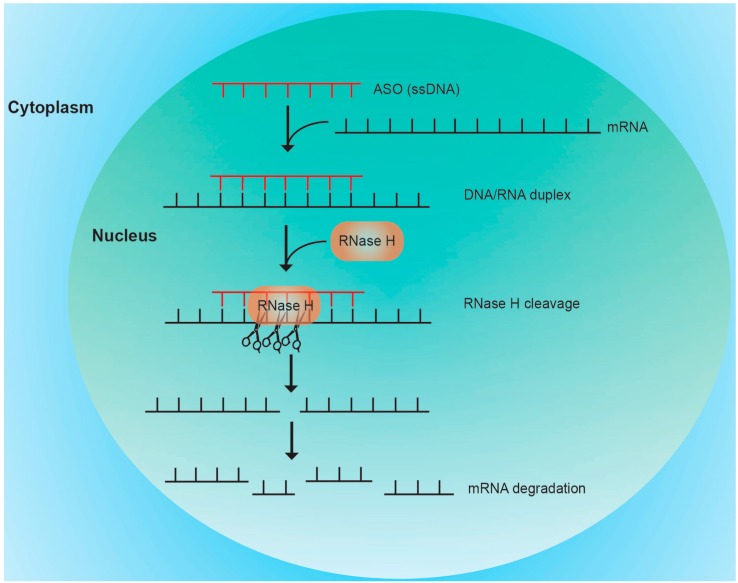
Schematic representation of ASO mediated gene silencing via RNase H cleavage. Upon delivery into the cell, ASO can traverse into the nucleus and binds to its complement in the target mRNA. The DNA/RNA hybrid is recognized by RNase H which cleaves the target mRNA. Cleavage by RNAse H is not absolute site specific and occurs within a small region of several nucleotides of the DNA/RNA duplex. After RNA cleavage, the ASO is believed to be reused for novel DNA/RNA hybrid formation.

Most commonly, ASO binding to the RNA is followed by induction of RNase H activity resulting in RNA degradation (Figure 2). The process takes place in the nucleus where endonuclease is ubiquitously expressed as shown for oocytes from *Xenopus laevis* [60]. Early findings have suggested that translocation of ASO to the nucleus occurs by passive movement across the nuclear pore [61]. The role of transporters, like microRNA transporter exportin-1 (Exp1), for clearance of ASO from the nucleus was recently suggested [62]. Accumulation of the ASO to the nucleus can also be achieved by fusion to proteins or inclusion to carriers suggesting that an active nuclear complex transport mechanism can take place [63,64]. After entering the nucleus, the formation of a DNA-RNA duplex stimulates RNase H activity. Hydrolysis of the 3′-*O*-P-bond of the RNA is observed, followed by degradation of the RNA [65]. The specificity of the RNase H binding to its target is not completely understood. Recognition of RNase H substrate involves interaction with 2′-*O*-hydroxyl groups of RNA [66]. X-ray crystallography studies imply that DNA-RNA hybrids show a characteristic curvature that is likely recognized by RNase H [67]. As compared to the siRNA mechanism (see next chapter), the specificity of the RNase H mediated cleavage within the RNA target is somewhat broader. A preferred cleavage has been observed within a region of 8–12 nucleotides from the 5′-RNA-3′-DNA terminus of the duplex with a high preference for GU sequence motifs. A minimal hybrid length of about 6 nucleotides seems to be required for RNase clevage [68]. Of note, as ASO is released from the RNase H complex after cleavage, a repeated use of the ASO molecule after RNA cleavage has recently been suggested both *in vitro* and *in vivo* corroborating that the ASO induced inhibition mechanism is highly efficient [69].

### 3.2. Nucelotide Modification

**Figure 3:**
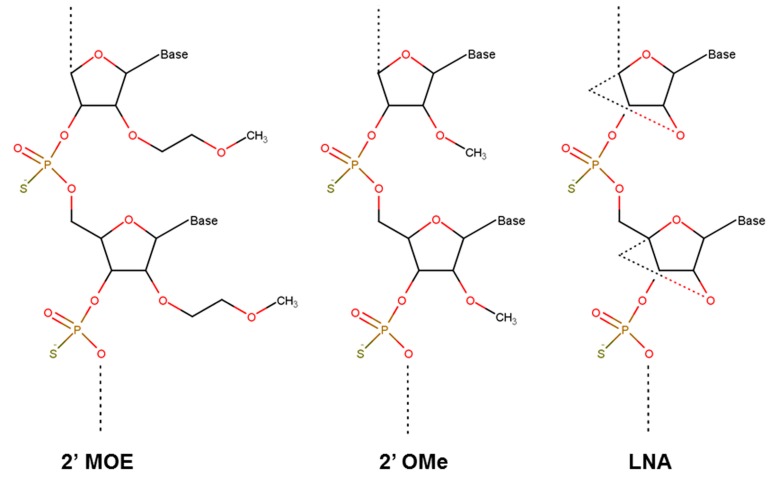
Nucleotide modifications frequently used for gene silencing. Modifications of nucleotides mostly involve the 2′ position of the ribose sugar as shown here for 2′-*O*-methoxyethyl (2′-MOE), 2′-*O*-methyl (2′-OMe), and 2′-*O*, 4′-*C*-methylene linked bicyclic ribofuranosyl modification (locked nucleic acids, LNA). Also note the phosphorothioate (PS) linkages in all molecules, where a non-bridging oxygen is replaced by sulfur, increasing its resistance to enzymatic hydrolysis.

One major obstacle of oligonucleotides is their rapid degradation in serum and cells after delivery. Promising therapeutic oligonucleotides were derived after steadily improvement of their stability and bioavailability achieved by chemical modifications of the nucleotides (Figure 3). Most modifications target the phosphodiester bond and the 2′-position of the ribose sugar. These variations have different effects on the corresponding duplex stability and nuclease resistance. One of the first nucleotide modifications used to stabilize oligonucleotides for *in vivo* delivery was successfully replacing the non-bridging oxygen in the phosphodiester backbone with sulfur to obtain a phosphorothioate deoxynucleotide and phosphorothioate (PS) modifications [70,71]. PS modified nucleotides have been found to predominately accumulate in the nucleus and induce formation of nuclear bodies believed to be important for antisense mechanism [72]. A pharmacokinetic (PK) benefit was observed due to binding of oligonucleotides to plasma proteins which decreased renal clearance and increased the half-life to about 1–3 days [73]. On the other hand, PS modifications decrease the melting temperature (Tm) as shown for various oligonucleotide combinations by roughly 8 °C per strand [74]. As a consequence, the affinity of the PS modified oligonucleotide to its target decreases as well. A so called second generation of ASO was developed to increase the affinity of the oligonucleotide to the mRNA and also to further improve the resistance to cellular nucleases and decrease cellular toxicity and side effects. The most significant chemical modifications to address these issues were the addition of 2′-*O*-methyl (2′-OMe) and 2′-*O*-methoxyethyl (2′-MOE) residues to the 2′-position of a ribose sugar [75]. 2′-OMe nucleotides can increase the Tm roughly by 1 °C per substitution [76]. Oligonucleotides covering these alkyl modifications show high stability to nucleases, increased affinity to target mRNA, lower toxicity, and an improved lipophilic character favorable for lipid bilayer diffusion [77]. The plasma half-life of ASOs in patients can be increased to around four weeks [78]. One drawback of alkyl-modified oligonucleotides is that the duplexes formed with target RNA have a decreased ability to induce RNase H activity [79]. The addition of locked nucleic acid (LNA) is amongst the most recent ASO modifications used for improvement of therapeutic oligonucleotides. LNAs show a modification of the ribose sugar at the 2′- and 4′-positions by means of a linkage with a methylene residue [80]. The formation of the methylene-bridge enhances the affinity of the oligonucleotide to the target RNA, strengthens the stability of the duplex, increases the lipophilic character, and improves the resistance to nucleases [81]. Introduction of LNA can increase Tm by up to 4 °C per substitution [82]. Kurreck and colleagues described a 10-fold increase in stability of LNA in comparison to unmodified oligonucleotides, which corresponds to a half-life of about 15 h *vs.* 12 h for 2′-OMe variations and 10 h for PS modifications [83].

### 3.3. ASO Gapmer

**Figure 4:**
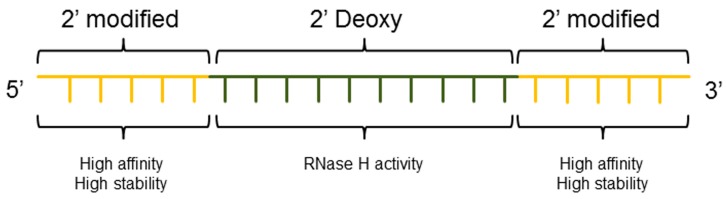
Gapmer. Current generations of ASO are frequently represented by a gapmer of typically 20 nucleotides with predominant phosphorothioate (PS) linkages. The central domain is composed of 8–12 deoxynucleotides that bind RNase H and induce efficient cleavage of the mRNA. At both ends of the gapmer (wings), 2–5 nucleotides having 2′ modifications, typically 2′-MOE and 2′-OMe, are inserted. The wings improve overall resistance of ASO to nucleases and increase the affinity to the target mRNA.

The development of chimeric ASO sequences called “gapmer” having a central phosphorothioate region of 8–12 deoxynucleotides responsible for binding of RNase H and flanked by 2′-alkyl-modified nucleotides on both sides to increase stability (Figure 4), was essential for further improvement of therapeutic oligonucleotides [84]. Currently, most advanced therapeutic ASO platforms in the clinic contain a nucleotide modification of 2–5 bases on both ends of the oligonucleotide that have excellent stability and allow efficient RNase H cleavage. Modification of nucleotides have also been used to develop dsASO where the sense strand is chemically modified in a way that it is more susceptible to hydrolysis by phosphodiesterases leaving the stable antisense gapmer for inhibition [85]. Besides the molecular impact of the most advanced ASO platforms for gene silencing, off-target effects exerted by the oligonucleotide have to be considered. Off-target effects can result in significant toxicity and frequently associate with the class of nucleotide modifications rather than with a specific sequence. Broadly, hybridization-dependent, e.g., due to binding at off-target RNA, or hybridization-independent, e.g., cytoplasmic granule accumulation or pro-inflammatory effects, can be distinguished [86].

The previously approved drug mipomersen features many of the above described chemical arrangements [15,87,88]. Mipomersen consists of a 20-base 2′-OMe gapmer having 5-mer “wings”. It belongs to the group of second generation ASOs and binds to a 20 nucleotide sequence of the human apolipoprotein B100 (apoB100) mRNA. In a recent placebo-controlled phase 3 clinical study the LDL concentration was found to be decreased by about 25% in the treatment group as compared to about 3% in the placebo group after having received 200 mg of ASO by weekly subcutaneous injections [89]. The most common adverse effects were noted in follow-up reports to be related to the injection site. Flu-like symptoms, increased ALT levels, and steatosis were also observed [90,91,92,93]. Alternative dose regimens of mipomersen have been suggested for long-term studies [94].

## 4. RNAi-Mediated Gene Silencing Using siRNAs

RNAi is a post-transcriptional regulatory mechanism that involves small double stranded RNA (dsRNA) for gene silencing in a sequence-specific manner. It is believed that RNAi might have arisen as a defense mechanism against viruses and transposons, which usually involves dsRNA for propagation and replication. The first report of RNAi gene silencing was by the Nobel laureates Fire and Mello who described long, dsRNA in the nematode *Caenorhabditis elegans* [2]. Therapeutic application of siRNA in a liver disease mouse model was demonstrated in 2003 via Fas silencing [95]. In the past decade, RNAi has become an attractive tool for development of therapeutic gene silencing [96,97].

### 4.1. RISC Mediated Cleavage

**Figure 5:**
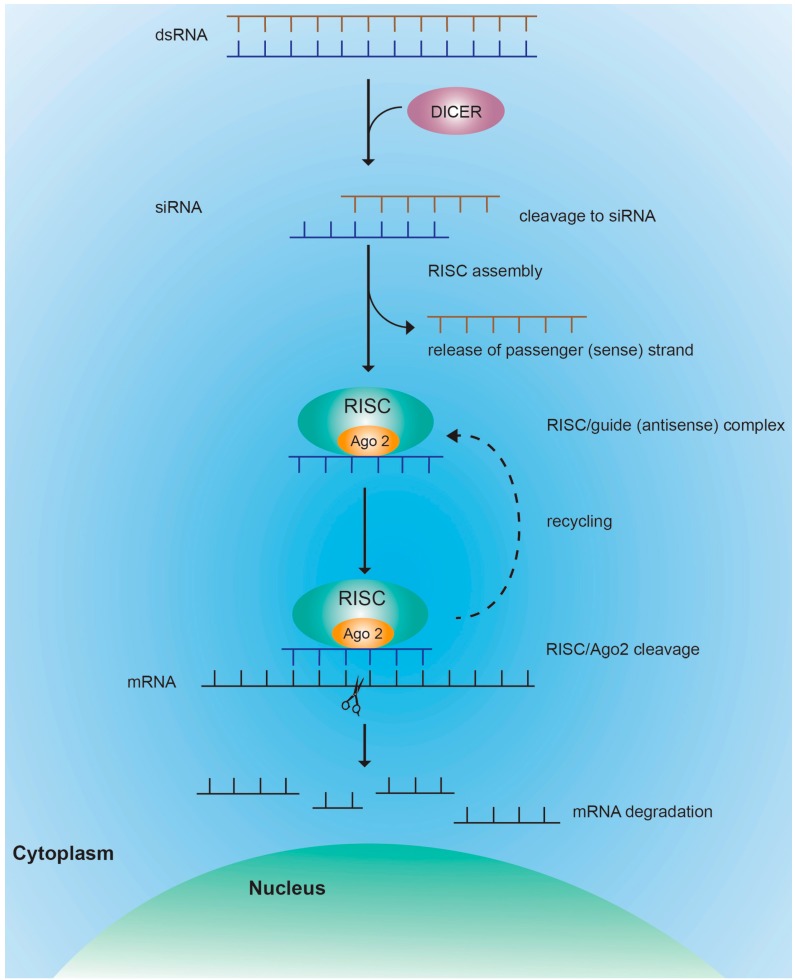
Schematic representation of siRNA mediated gene silencing. Long dsRNA is cleaved by enzyme Dicer into 20–30 bp siRNA, which consists of a passenger strand (sense) and a guide strand (antisense). Alternatively, a mature siRNA containing a 5′ phosphate and a two-nucleotide overhang at the 3′ end can be delivered to cells. The multiprotein enzyme complex RISC recognizes the siRNA in the cytoplasm, unwinds the duplex siRNA and incorporates the guide strand (antisense) while the passenger strand is released. RISC then uses the guide strand to specifically target mRNA. For perfectly matching targets, the endonuclease Ago 2 within RISC induces one cleavage that takes place at position 10 upstream of the 5′ end. After cleavage, the siRNA-RISC complex is recycled to target other mRNA.

Short dsRNA are classified as small interfering RNA (siRNA) and micro RNA (miRNA). A variety of RNA structures can be used to induce RNAi, including Dicer substrate RNAs or shorter siRNAs. In mammalian cells, siRNA is generated by cleavage of long dsRNA into smaller RNA molecules of ~20–30 bp in length (Figure 5) [98,99]. Dicer, a large, multidomain RNase III endonuclease enzyme, complexed with the TAR-RNA binding protein (TRBP), is responsible for this cleavage [100]. The resulting siRNA has a 5′ phosphate and a two-nucleotide overhang at the 3′ end [101]. This unique characteristics allow recognization by the enzymatic complex termed RNA-induced silencing complex (RISC). The ~670 kDa RISC complex is composed of Argonaute 2 (Ago 2), Dicer, and TRBP [102]. Each siRNA duplex consists of a guide (antisense) strand and a passenger (sense) strand. The strand with the lowest duplex stability at the 5′ end (guide strand) is incorporated into RISC [103]. Unwinding and release of the passenger strand is accomplished by Ago 2, which forms the catalytic core of the RISC complex [104,105]. RISC uses the guide RNA to locate the complementary RNA sequence leading to endonucleolytic cleavage of target mRNA by Ago 2. In contrast to ASO, there is predominantly one cleavage event that takes place at the nucleotide position 10 upstream of the siRNA 5′ end [106]. The mechanism by which RISC finds the target mRNA is not completely understood. The accessibility of the target mRNA (e.g., by absence of secondary and tertiary structures) directly correlates to cleavage efficacy [107]. The guide RNA/protein complex is a multiple turnover enzyme and is recycled after cleavage allowing the initiation of an enzymatic cascade. Only one or few RNA molecules within the target cell are sufficient for cleavage which explains the remarkable efficiency of RNAi-mediated silencing [108,109]. Continuous inhibition of target mRNA has been observed in non-dividing cells, such as hepatocytes, for a period of 3–4 weeks on siRNA application [110]. Of note, for the liver it has been suggested that siRNA can be exchanged between cells, partially mediated by shuttling of exosomes, even without direct cell-cell contact which may further potentiate the efficacy [111]. The RNA loading and activity of the siRNA mechanism typically takes place in the cytoplasm; however, a nuclear localization of several siRNA components has recently blurred this view [112].

### 4.2. Modification of siRNA

While RNAi is a popular gene silencing mechanism, there are obstacles that need to be overcome to exploit its properties for genetic manipulation of diseases. Like small molecule and monoclonal antibody modalities, understanding the PK and biodistribution of oligonucleotides is essential for its therapeutic application. Unmodified, naked siRNA has a very short plasma half-life (up to few minutes) making it highly susceptible to degradation [113]. Chemical modifications of nucleotides that enhance stability have helped in overcoming these problems (Figure 3). In principle, nucleotides that were used for generation of ASO (see previous chapter) can also be used for siRNA [114,115]. While it was shown that larger substituents at the 2′ position are frequently used in ASO, 2′-*O*-variations counteract with the silencing activity of siRNA due to limited compatibility with Ago2 [116,117]. Care must be taken since such modifications can reduce siRNA specificity and may result in higher toxicity. While the passenger strand can be modified almost completely, only a few positions of the 3′ end and middle region of the guide strand can be changed without affecting the gene silencing ability of siRNA.

Critical problems with RNAi gene silencing are off-target effects, e.g., unintended downregulation of mRNA transcripts outside the target gene. This is caused due to partial sequence complementarity of sense or antisense siRNA strands to non-target mRNA. Off-target silencing of a large number of genes on treatment with siRNA was demonstrated by microarray technology [118]. Genome-wide expression profiling in mammalian cells revealed siRNA-specific rather than target-specific signatures, causing silencing of mRNAs having as few as 11 contiguous nucleotides of identity [118]. Furthermore, studies of siRNA transfected cells showed induction of toxic phenotype as a consequence of off-targeting [119]. Hence, stringent siRNA screening is mandatory to determine tolerable levels of off-targeting without affecting the phenotype. Of note, chemical modification of siRNA can reduce off-target effects, e.g., the addition of a methyl group to the 2′-position of the ribosyl ring in the siRNA guide strand can reduce off-target effects by 80% without reducing on-target silencing [120].

Introduction of dsRNA and ssRNA can activate innate immune responses via interactions with Toll-like receptors (TLRs) present on the cell surface leading to stimulation of pro-inflammatory cytokines and partial interferon response, namely via TLR3 and TLR7/8, respectively [121,122]. Such immune stimulatory effects are highly dependent on features of the siRNA, especially when the length exceeds 30 bp. The sequence, nucleotide modifications, and chemical conjugations also play a role. A particular sequence motif (5′-GUCCUUCAA-3′) was identified within siRNA which seems to be recognized by TLR7 of dendritic cells to activate immune responses [123]. Though stimulation of immune responses could be beneficial in some clinical application, it raises serious concerns for the safe use of RNAi in therapeutics. Sequence modifications including 2′-F, 2′-OME, and 2′-H substitutions in anti-HBV siRNA have been shown to abolish cytokine induction, thereby reducing immunogenicity [124]. Adverse effects observed in human studies that are caused by new generation siRNAs are generally mild; however, oral corticosteroids, histamine receptor (H1 and H2) blockers, and paracetamol are sometimes given to the patients shortly prior to administration [125].

## 5. Delivery of Oligonucleotides to the Liver: An Attractive Target for Therapeutic Oligonucleotides

It has long been noted that radiolabeled oligonucleotides, when systemically applied to the human body, are greatly accumulated in the liver. While high oligonucleotide accumulation is also observed in the kidney, other organs, like the spleen, heart, pancreas, and the brain show far lesser concentrations [126]. The liver has a major role in the human body with numerous functions, including the homeostasis of glycogen, decomposition of red blood cells, synthesis of important plasma proteins, hormone production, and first entry level of detoxification. Currently, therapeutic oligonucleotides are subjected to clinical trials for prevention of various liver disease [11,87,127]. Liver cell uptake and subcellular distribution are essential for the pharmacological action of the oligonucleotides. Upon systemic administration following intravenous (i.v.), subcutaneous (s.c.), or intraperitoneal (i.p.) injection, oligonucleotides must overcome several biological barriers, like degradation by nucleases in serum and cells, renal filtration, rapid clearance via reticuloendothelial system (RES), endothelial cell barriers, and diffusion through extracellular matrix before entry into the target organ *i.e.*, the liver.

The liver is a well-perfused organ with an endothelium that acts like an accessible doorway (sinusoidal sieve) for larger moieties. Blood from the intestine and spleen is passed to the liver by the hepatic portal vein. The portal blood and the arterial blood, which transports the oligonucleotides after i.v. administration, mix in the hepatic sinusoids before leaving via the hepatic vein. Lobules having hexagonal shape represent the organizational unit structure of epithelial hepatocytes, arranged into cords that are surrounded by vascular sinusoids. While a basal lamina is missing, relatively large fenestrations (100–200 nm diameter) between the endothelial cells allow for increased extravasation into the parenchyma in a unique architecture termed the space of Disse. Besides hepatocytes that represent about 80% of the liver parenchyma, other types of cells, prominently Kupffer cells, stellate cells and various cells of the immune system, can also take up significant amounts of the oligonucleotides. Depending on the dose, up to 80% of oligonucleotides can be found in non-parenchymal cells of the liver [128]. The timing of oligonucleotide administration is also an important factor. Delivery with slow infusion of ASO as opposed to bolus injection led to increased concentration in the liver [129]. It is therefore of interest that a strong pharmacokinetic/pharmacodynamic (PK/PD) relationship has been observed for most therapeutic oligonucleotides that are in late stage clinical trials, including mipomersen [130].

### 5.1. Biological Barriers for Liver Targeted Oligonucleotides

Oligonucleotides can circulate freely or in a non-covalently bound form associated to plasma proteins. Following different injection routes (i.v., s.c., and i.p.), the i.v. administration resulted in much higher oligonucleotide levels in the liver, at least for a 2′-OMe ASO targeting the dystrophin gene [131]. In blood, the adsorption to various types of proteins, termed opsonins which mediate recognition and uptake by RES, has to be avoided. In a free and chemically unmodified form, renal filtration and clearance of oligonucleotides is extremely high. A hydrodynamic diameter of <5–6 nm is associated with renal clearance within minutes. Oligonucleotide-protein complexes pass across the vascular endothelial barrier and are transported by blood throughout the body, particularly to the liver. It has been shown that PS modifications can highly improve the binding efficiency to plasma proteins. The ASO “ISIS 2302” directed against ICAM-1 has been shown to attach to more than 97% of plasma proteins with albumin being the most frequent favoring delivery to the liver [132]. Without further modification, as shown for ASO that were linked to other tissue-specific and cell-penetrating peptides, the majority of the oligonucleotides are found in the liver [133,134].

The next barrier of the oligonucleotides is represented by the plasma membrane, e.g., of the hepatocyte. The high molecular weight and the overall negative charge of the oligonucleotide impede uptake by cells. While *in vitro* various transfection agents can compensate such shortcomings, these are mostly toxic and not in line with human use and clinical studies. Uptake of oligonucleotides by endocytosis, micropinocytosis, and lately also by direct translocation have been discussed [135]. Like other biological macromolecules, the oligonucleotides can enter the target organ via receptor-mediated endocytosis or by other pathways [134,136]. Coated pits invaginate into the cytoplasm and pinch off to form clathrin-coated vesicles followed by sequential vesicular trafficking from early endosome to late endosome and lysosomes [137]. However, clathrin-independent pathways of oligonucleotide uptake have also been observed. The PS-mediated uptake of naked siRNA follows a caveosomal mechanism and a direct translocation has been observed for peptide based nanoparticles [138,139]. Covalent conjugation of ASO and siRNA with cell-penetrating peptides (CPPs) has been established for effective delivery to mammalian cells and appropriate subcellular target delivery [140,141,142]. *In vivo* delivery of proteamine-antibody fusion proteins that bind siRNA could specifically deliver the oligonucleotide to HIV-1 envelope expressing cells [143]. Conjugation of siRNA with aptamers binding to PSMA, a cell-surface receptor overexpressed in prostate cancer cells, has also resulted in cell-type specific delivery *in vivo* [144].

One final biological barrier is represented by endosomal escape. The mechanism of intracellular trafficking is however poorly understood and needs further research. Receptor-ligand complexes that have been endocytosed to the early endosomes behave in one of the two ways: they may return to the plasma membrane by vesicular transport or may be transported further to the lysosome, where they are degraded by hydrolytic enzymes. For a productive pathway, the oligonucleotide must reach its site of action in the nucleus or cytoplasm by exiting the endosomes. The productive pathway can account for less than 20% of the total oligonucleotide delivered to liver tissue [129]. In the mouse hepatocellular carcinoma cell line MHT, the productive siRNA pathway involves vesicles which are dependent on adaptor protein subunit AP2M1 but independent from clathrin or caveolin [145]. ASO that are bound to a conjugate via disulfide linkages can be released from carrier during endosomal exit due to weak intermolecular interaction that are prone to cleavage in acidic endosomal environment [146]. To obtain high accumulation of the therapeutic oligonucleotide inside the nucleus, there is need of an active transport mechanism. PS modified ASO were observed to shuttle between nucleus and cytoplasma via an active ATP-dependent mechanisms [147]. Conjugation of ASO with negatively charged liposomes can also result in high nuclear delivery [148]. Detailed understanding of these biological processes has helped design specific strategies in overcoming such barriers, e.g., conjugating oligonucleotides with endosomal release signal peptides or nuclear localization signal peptide [149,150]. Also, acid-labile maleamate bonds have been used to escape endosomal arrest after apoB targeting [151].

### 5.2. Molecular Strategies for Specific Liver Targeting

In contrast to siRNA, most ASO of newer generations do not need formulations to exert robust antisense effects. However, in a side by side comparison of siRNA and ASO that were designed to target the tumor suppressor gene, phosphatase and tensin homologue (*PTEN*) unmodified and LNP formulated oligonucleotides were investigated after single i.v. administration [152]. Here, a significant downregulation of *PTEN* (>75%) was achieved in the liver by both oligonucelotides only after a LNP formulation was used. As estimated by determination of the integrity of both oligonucleotides in the liver (e.g., presence of 5′ phosphate group essential for RNAi), siRNA seems to benefit most from LNP formulations. Basically, two broad approaches for stabilization of the delivery can be distinguished. The first involves incorporation of oligonucleotides into lipid or polymer nanocarries, like the shielding agent polyethylene glycol (PEG), allowing increased stability [153] while the second involves molecular conjugates where oligonucleotides are linked to ligands that binds to specific cell surface receptors with high affinity [154,155,156]. Additionally, dendrimers consisting of branched polymers, usually in the range of 10–100 kDa, have been reported for improved oligonucleotide uptake [157]. The protection by PEG involves lower unwanted protein binding and lower stimulation of the innate immune system [158,159]. PEG is used to mask the membrane disrupting activity via acid-cleavable carboxylated dimethyl maleic acid (CDM) [160]. The most advanced delivery platform for systemic administration is lipid nanoparticles (LNPs), with mipomersen as one prominent example. LNPs bind apolipoprotien E (apoE) in the circulation and the apoE complex facilitates receptor-mediated uptake by hepatocytes [161]. Stable nucleic-acid-lipid particle (SNALP) that consist of a lipid bilayer containing a mixture of cationic and fusogenic lipids including PEG was used for siRNA delivery [124].

Oligonucleotides, having per se an inherent preference for accumulation in the liver, have been further subjected to refined, liver-specific delivery strategies that are thought to increase efficacy, e.g., for use of lower doses. Carbohydrate-based-ligands, e.g., galactose [162], galactose derivative *N*-acetylgalactosamine (NAG) [163], and lactose [164], are frequently employed. Conjugation of siRNA to GalNAc, a highly efficient ligand for the asialoglycoprotein receptor (ASPGR) is the leading carbohydrate-siRNA conjugate in clinical development. The ASGPR is almost exclusively found on hepatocytes where it is located at the basolateral membrane, directly facing the bloodstream. The number of ASGPR has been estimated to be ~500,000 copies/cell [165]. The receptor is highly conserved and binds serum glycoproteins followed by receptor-mediated endocytosis [166]. Conjugation of the 3′ terminus of siRNA with three molecules of *N*-acetylgalactosamine (GalNAc) resulted in sustained gene silencing after weekly administration for over 9 months with no adverse effects in rodents [163]. A s.c. delivery of GalNAc-siRNA conjugates seems to pose advantage over LNPs which had to be delivered i.v. to elicit favorable RNAi-mediated therapeutic effects [163]. However, s.c. administrated LNP can also give robust inhibition when intermediate size particles (~45 nm) or GalNAc were incorporated [167]. A co-administration strategy of GalNAc-modified DPC polymer and cholesterol conjugated siRNA improved efficacy to about 500-fold over single use of cholesterol siRNA and resulted in 90% reduction in mice and non-human primates [168]. Of note, for efficient inhibition to take place intracellular cleavage seems to be necessary that removes conjugates [169,170].

## 6. Clinical Studies Employing ASO Directed against Human TTR

Antisense compounds targeting human TTR were recently developed and transferred to clinical trials by ISIS Pharmaceuticals (www.ttrstudy.com). ISIS Inc. is targeting a variety of diseases, most notably by conducting advanced clinical trials in the fields of cardiovascular, metabolic, neurodegenerative diseases, and cancer. ISIS 420915 (TTR_Rx_) is a second-generation ASO gapmer directed to human TTR, having wings of 2′-MOE-modified ribonucleotides and PS linkages. The sequence of the ASO is fully complementary to a region within the 3′ untranslated region (3′ UTR). A set of 400 TTR ASO compounds was originally screened in the human hepatoma cell line HepG2 and also evaluated in a TTR Ile84Ser transgenic mouse model [171]. Eight compounds were further selected for analysis in non-human primates and shown to suppress serum TTR. The anti-TTR ASO was effective in rodents [172,173]. A Phase 1 trial of ISIS 420915 was conducted in healthy volunteers as a blinded, randomized, placebo-controlled, dose-escalation study designed to assess the safety and PK profile. After four weeks of dosing (50 mg to 400 mg), ISIS 420915 caused rapid and significant dose-dependent reduction of serum TTR levels. Reductions of up to 96% (average around 75%) were observed as compared to the pre-treatment levels. From the five doses studied, the 300 mg dose was chosen for the subsequent trials. The compound was generally well tolerated in all subjects and safety and tolerability profile supported the progression of ISIS 420915 directly to Phase 3 studies.

In December 2012, a randomized, double blind, placebo controlled, international Phase 3 study was started with the collaborator GlaxoSmithKline. After an initial loading period (three s.c. injections on alternate days in the first week), ISIS 420915 was self-administered at home with one s.c. injection per week for another 64 weeks. About 200 patients with mild stage neuropathy (stage 1 and stage 2) are envisaged to be enrolled in the study. FAP patients from various countries, including United States, France, Germany, Italy, Portugal, United Kingdom, Spain, and Argentina have already entered the trial. The mutations of the FAP patients represent a total of 37 different TTR mutations, with Val30Met being most prevalent. The current primary endpoint of the study is the efficacy as determined by nerve sensory measurements (modified Neuropathy Impairment Score mNIS + 7) as well as by quality-of-life (Norfolk Quality of Life Diabetic Neuropathy questionnaire). mNIS + 7 is an evaluation of muscle weakness, sensory/autonomic function, and nerve conductance, where the progression of neuropathy in FAP patients usually leads to an increased score over time. As a secondary endpoint of the study, the levels of TTR and retinol binding protein 4 (RBP4) will be assessed. Evidence regarding the efficacy of ISIS 420915 to treat FAP disease is not yet available. Final data are awaited not before November 2016 (the final data collection date for primary outcome measure). However, the continuing, blinded safety analysis of the Phase 3 study shows that the injection site reactions were predominantly mild and infrequent, occurring in only about 1% of all injections.

In 2015, ISIS Pharmaceuticals reported first results from an unblinded study, termed open-label extension (OLE) study. FAP patients that have completed the Phase 3 study were enrolled in this study in which all patients receive ISIS 420915 and no placebo group is implemented. A first group of patients (*n* = 13) at month three of the OLE study showed a reduction of serum TTR level up to 92 percent (median reduction of 78 percent) compared to the baseline at the Phase 3 study suggesting that ISIS 420915 is highly effective to reduce TTR levels of the FAP patients. Further clinical data with regard to the efficacy of FAP treatment are awaited soon. FAC patients were also recently included in study of ISIS 420915 [174].

### Alternate Clinical Target of TTR ASO

In an attempt to further improve the TTR ASO, ISIS developed a GalNAc conjugation of the oligonucleotide and evaluated the efficacy in the Ile84Ser transgenic mouse model [175]. As compared to the unconjugated ASO, the potency of TTR downregulation was increased by about 10-fold suggesting that a significant reduction of the ASO dose was achieved by the improved targeting of hepatocytes. In addition, recent experimental evidence obtained by ISIS using the standard TTR ASO platform suggests that the anti-TTR therapy could play a role for the treatment of type 2 diabetes [176]. It has long been known that RBP4 levels are increased in most insulin-resistant humans. In two insulin-resistant models, obese mouse (*ob*/*ob*) and the high-fat diet mouse model, application of TTR ASO resulted in a decrease by 80%–95% of circulating RBP4 concomitant with the decrease of TTR. Also, insulin level were found to be decreased by 30%–60% following TTR silencing. The finding suggests that reduction of the RBP4 level by the TTR ASO could improve insulin resistance opening a wider field of clinical application for this therapeutic oligonucleotide [177].

## 7. Clinical Studies Employing siRNA Directed against Human TTR

Alnylam Pharmaceuticals, dedicated to the development and clinical assessment of siRNA in various diseases, such as genetic, cardio-metabolic and hepatic infection disease, is currently the leader of siRNA-based approaches to silence human TTR (www.alnylam.com). A multicenter, randomized, single-blind, placebo-controlled Phase 1 study was reported for one of Alnylam’s most promising TTR siRNA, termed ALN-TTR02 (Patisiran) [178]. ALN-TTR02 is a second generation siRNA, formulated with lipid nanoparticles that targets the 3′ UTR of the human TTR mRNA. ALN-TTR02 (doses of 0.01 to 0.5 mg/kg) was applied to subjects via i.v. infusion. On administration of the compound no drug-related serious adverse events and significant changes in hematologic, liver, or renal measurements were observed. Mild-to-moderate infusion-related reactions (IRRs) occurred in 7.7% of the participants. Antibodies to the pegylated lipid component of the drug were not detected in patients. Reductions in TTR serum levels at doses of 0.15 mg/kg to 0.3 mg/kg were observed and ranged from 82.3% to 86.8%, with reductions of 56.6% to 67.1% at day 28. These reductions of TTR were associated with a reversible decline in levels of RBP and vitamin A that were however not found to be adverse. Of note, to reduce the risk of lipid-related reactions, an oral premedication (dexamethasone, paracetamol, H1 and H2 blockers) was given prior to Patisiran infusion.

In a multi-center, dose-escalation Phase 2 study of ALN-TTR02 the safety and tolerability of the compound was further evaluated in FAP patients (*n* = 29). Patients received two doses of ALN-TTR02 in 5 cohorts with doses ranging from 0.01 to 0.30 mg/kg, using either a once-every-four-week or once-every-three-week dosing regimen. Multiple doses of ALN-TTR02 were found to be generally safe and well tolerated with mostly IRRs that were observed in 10.3% of patients [179]. No IRRs were however reported in 12 patients who received 0.30 mg/kg once every three weeks. Infusion times of more than 70 min seemed to be favorable to prevent adverse reactions including IRR. The multiple doses of ALN-TTR02 resulted in a rapid and sustained reduction of serum TTR levels, with mean reduction levels of >85%.

A recent summary of a 12-month data-cut derived from the extended open label Phase 2 study (OLE) was reported by Alnylam. The data suggest a mean 2.5 point decrease in mNIS + 7 (modified Neuropathy Impairment Score) observed in a portion of the FAP patients (*n* = 20) as compared to historical data sets of untreated FAP patients with similar baseline characteristics. In this OLE study, ALN-TTR02 was administered once every 3 weeks at a dose of 0.3 mg/kg. These first results are encouraging and suggest for the first time that TTR knockdown may halt FAP disease progression. Other clinical measurements, including quality of life (QOL), a 10-m walk test to evaluate mobility, and modified body mass index (mBMI) were found unchanged in this preliminary analysis. For a more detailed analysis of the therapeutic efficacy, Alnylam has started a randomized, double-blind, placebo-controlled Phase 3 study of ALN-TTR02 (APOLLO) in 2013. The primary endpoint of the study is the difference in the change in mNIS + 7 between ALN-TTR02 and placebo treated FAP patients at the end of the study (month 18). The trial is designed to enroll 200 FAP patients (stage 1 or stage 2). First data are awaited towards the end of 2016.

### GalNac Modification of TTR siRNA

A second siRNA compound, termed ALN-TTRSC (Revusiran), also directed against human TTR, was recently developed by Alnylam. In ALN-TTRSC the sense strand of the siRNA was covalently linked to GalNAc. Due to Alnylam’s modified siRNA platform chemistry a lipid formulation does not seem to be necessary to stabilize the GalNAc oligonucleotide. ALN-TTRSC is administered by s.c. injection to patients. A premedication procedure prior administration of ALN-TTRSC is not reported. The compound has already been subjected to Phase 1 and Phase 2 clinical trials. Preliminary results of the Phase 2 study have been reported for 14 FAC and 12 SSA patients. Revusiran was administered initially as daily s.c. doses for five days and then once weekly for five weeks at doses of 5.0 mg/kg or 7.5 mg/kg. The data indicate that ALN-TTRSC results in a high serum TTR knockdown of up to 98.2% while the compound is well tolerated with only mild adverse reactions (IRR in 23% of patients). The knockdown was observed in FAC and SSA patients corroborating that the compound target mutant and wild type TTR with equally high efficiency. As expected from the short treatment time of 5 weeks, no other significant changes of the disease could be determined in this study. In December 2014, a randomized, double-blind Phase 3 multicenter study of ALN-TTRSC (ENDEAVOUR) was started by Alnylam for therapy of FAC. Patients received weekly s.c. injections of 500 mg ALN-TTRSC or placebo. Here, a walk test to assess the physical fitness of the patients is the primary endpoint and will be compared between groups at the end of the study at month 18. The presumed final data collection date for primary outcome is scheduled for 2018.

## 8. Concluding Remarks

Over the past decades there has been an exponential growth in the pursuit of exploiting the properties of oligonucleotides in therapy. Oligonucleotide-mediated therapy has become a powerful technique for ablation of targeted gene expression in mammalian cells. As observed in various preclinical studies, the liver is an excellent target for therapeutic oligonucleotides and, besides mipomersen, various oligonucleotides addressing different liver diseases are now studied [11]. Targeting the TTR gene by ASO and siRNA is now under evaluation in advanced clinical studies and could be highly valuable to further confirm the clinical feasibility of the concept (Table 1). From both the clinical and molecular perspective, stunningly high TTR downregulation rates of around 80% have been achieved in the patients over several months of treatment. Presently, observation times of more than one year have documented only minor adverse effects, mostly related to the injection sites while the TTR silencing seems to be robust. The anti-TTR oligonucleotides are administered by systemic routes in a routine interval of one or three weeks corroborating that such regimen allows for a stable gene inhibition, even when a highly expressed gene, like TTR, is targeted. Once inhibition was established in patients, TTR expression only gradually increased after last injection suggesting that the molecular mechanism of inhibition is quite sustained over a period of 1–3 weeks, outperforming the short half-life of the TTR protein. As discussed here and elsewhere [6,97,136], the therapeutic oligonucleotides have to pass several biological barriers to finally target TTR mRNA which is thought to take place in the cytoplasm (RNAi) or nucleus (ASO). Stoichiometric calculations of the effective number of molecules needed per cell are limited. It is known that one molecule of the oligonucleotide can be reused for several rounds of inhibition [69,109] suggesting that a relative low number of molecules associated to the enzymatic machinery, either RNase H or RISC, are sufficient for mRNA silencing in hepatocytes. The liver of an adult male (1.5 kg) harbors ~2 × 10^11^ hepatocytes [180]. From the current doses (300 mg ASO and 0.3 mg/kg siRNA) applied to FAP patients it can be calculated that ~10^18^ to 10^19^ oligonucleotides are administered. Further molecular knowledge from ongoing human clinical trials as well as from preclinical studies will be valuable to identify and optimize the rate limiting steps of oligonucleotide delivery for human administration. Although oral administration of oligonucleotides seems to be far less efficient, such a route of administration may allow to augment the comfort of patients when using improved formulations [181].

**Table 1:** Therapeutic oligonucleotides currently used in clinical trials of TTR amyloidosis.

	ISIS-TTR_Rx_ (ISIS Pharmaceuticals)	ALN-TTR02 (Alnylam Pharmaceuticals)	ALN-TTRSC (Alnylam Pharmaceuticals)
mRNA target	3′ UTR	3′ UTR	3′ UTR
Oligoncleotide	DNA	RNA	RNA
Nucleotide modification	PS, 2′-MOE	LNP	GalNAc
mRNA degradation	RNase H-dependent	RISC	RISC
Primary site of action	nucleus	cytoplasm	cytoplasm
Administration	subcutaneous	systemic infusion	subcutaneous
Premedication	No	Yes	No
Study start-estimated completion	12/2012–11/2016 ^a^	11/2013–01/2017 ^b^	12/2014–12/2018 ^c^
Dosing	weekly 300 mg (3 doses first week)	0.3 mg/kg every 3 weeks	weekly 500 mg (5 doses first week)
Serum TTR knockdown	~80% ^d^	~80% ^d^	~80% ^d^
Disease	FAP, FAC, SSA	FAP	FAC

^a^ NCT01737398; ^b^ NCT01960348; ^c^ NCT02319005; ^d^ preliminary report.

As anti-TTR oligonucleotides are subjected to turnover, the compounds have to be taken lifelong in order to maintain a reduction or halt of the disease burden. Off-targeting effects, immune responses, and efficacy will have to be carefully monitored during prolonged administration. On the other hand, such long periods of TTR downregulation may give unprecedented insights into molecular mechanisms of the disease as compared to previous therapies that could not achieve reduction of wild type TTR synthesis [43,45]. It will be interesting to learn whether overall reduction of TTR in the circulation (~80%) will be accompanied by a regeneration of already diseased tissue, e.g., by downsizing of established plaques in the heart or elsewhere. Such processes might take several years and direct measurements of respective clinical endpoints may be included in long-term follow up studies. Downregulation of TTR, being the carrier of retinol and thyroxine, does not seem to grossly impact physiological functions [26,27] suggesting that a preventive supplementation with vitamin A, if necessary at all, might suffice to compensate any deficiencies in human. This peculiarity of TTR biology has to be kept in mind when other gene targets are addressed by therapeutic oligonucleotides, since vast inhibition of other target genes could impair physiological function. However, as downregulation of the target gene is not complete, residual levels of target gene expression might be sufficient for physiological function. The upcoming translation of TTR oligonucleotide-mediated therapy to the clinic represents a crucial turning point in treating a wide array of diseases that were previously considered “undruggable”. While we have focused in this review on the benefits of ASO/siRNA for treatment of TTR-related disease, the scope of therapeutic oligonucleotides might however go beyond liver disease.

## Abbreviations

Ago 2: Argonaute 2
ALT: Alanine transaminase
ASO: Antisense oligonucleotide
ASPGR: Asialoglycoprotein receptor
CDM: Carboxylated dimethyl maleic acid
CPPs: Cell-penetrating peptides
ds: Doublestranded
FAP; FAC; SSA: Familial amyloid polyneuropathy/cardiomyopathy; senile systemic amyloidosis
GalNAc: *N*-acetylgalactosamine
i.p.: Intraperitoneal
IRR: infusion-related reactions
i.v.: Intravenous
kDa: Kilodalton
LDL: Low-density lipoprotein
LNA: Locked nucleic acid
miRNA: Micro RNA
NAG: N-acetyl galactosamine
ncRNAs: Non-coding RNAs
2′-OMe: 2′-*O*-methyl
2′-MOE: 2′-*O*-methoxyethyl
PD: Pharmacodynamic
PEG: Polyethylene glycol
PK: Pharmacokinetic
PS: Phosphorothioate
RBP: retinol binding protein
RES: Reticuloendothelial system
RISC: RNA-induced silencing complex
RNA: Ribonucleic acid
RNAi: RNA interference
s.c.: Subcutaneous
siRNA: Small interfering RNA
SNALP: Stable nucleic-acid-particle
TLRs: Toll-like receptors
Tm: Melting temperature
TTR: Transthyretin
